# Reduced *β*2GPI Inhibiting Glomerular Mesangial Cells VEGF-NO Axis Uncoupling Induced by High Glucose

**DOI:** 10.1155/2018/5484731

**Published:** 2018-07-18

**Authors:** Zhou Saijun, Li Xin, Wang Jie, Xiao Shumin, Liu Shuaihui, Yu Pei

**Affiliations:** Key Laboratory of Hormones and Development (Ministry of Health), Metabolic Diseases Hospital and Tianjin Institute of Endocrinology, Tianjin Medical University, Tongan Road No. 66, the District of Heping, Tianjin 300070, China

## Abstract

VEGF-NO axis uncoupling is an important pathogenesis for DN. Reduced *β*2GPI could play a part in VEGF signaling pathway and has a protective effect on diabetic vascular disease. This study investigates the effect of reduced *β*2GPI on glomerular mesangial cells VEGF-NO axis uncoupling induced by high glucose. Compared to control group, glomerular mesangial cell line HBZY-1 cells treated with high glucose expressed higher levels of VEGF mRNA and protein and produced more ROS but less NO. The related proteins related to VEGF-NO axis were assayed. High glucose could significantly increase the expression of the level of VEGFR2 and obviously increase phosphorylation of Akt and eNOS but significantly decrease the expression of GTP cyclohydrolase 1 (GCH-1), reducing the production of eNOS dimer. Both *β*2GPI and reduced *β*2GPI partly reverse these effects caused by high glucose. Reduced *β*2GPI had stronger effect than *β*2GPI. GCH-1 is the speed limit of tetrahydrobiopterin (BH4) synthesis enzyme. As the key part of eNOS cofactors, BH4 could partly restore eNOS dimer induced by high glucose. Our results indicated that high glucose could interfere with eNOS dimer formation. *β*2GPI and reduced *β*2GPI can partly reverse the VEGF-NO axis uncoupling by restoring the GCH-1 expression level and then promote eNOS dimer formation.

## 1. Introduction

Diabetic nephropathy is one of the common chronic complications of diabetes and has become the leading cause of end-stage kidney disease in western countries. As for china, the morbidity and the proportion of diabetic nephropathy in the chronic dialysis kidney disease are growing. According to large number of clinical and basic researches, hyperglycemia is the initiation factor contributing to renal injury [1]. However, even glucose and lipid metabolic disorders have been strictly controlled, and they cannot completely prevent the progression of diabetic nephropathy [2].

Recent researches indicate that VEGF-NO axis uncoupling with a specific pathologic characteristic of higher expression levels of VEGF and lower production of NO in kidney may be one of the important pathogeneses for DN [3]. In recent years several relevant evidences have revealed that VEGF-NO axis uncoupling is involved in the injury of glomerular endothelial cells, glomerular mesangial cells, and podocyte [4]. And glomerular mesangial cells' injury is considered as a key stage in the process of diabetic nephropathy. Because of its central position in the renal glomerulus, hypertrophy of mesangial cells influences extracellular matrix (ECM) protein synthesis and expansion, which can further result in renal fibrosis and glomerulosclerosis [5]. VEGF-NO axis uncoupling could cause intracellular ROS in mesangial cells and in diabetic kidneys. ROS generation appears to play a major role in mesangial ECM expansion, the major pathologic feature of diabetic nephropathy. Increases in oxidative stress can further increase the production of inflammatory cytokines such as NF-*κ*B, ICAM-1, IL-6, and TNF-*β*. And, likewise, an increase in inflammatory cytokines can stimulate the production of free radicals. So, VEGF-NO axis uncoupling in mesangial cells may play an important part in the pathogenesis of diabetic nephropathy.

*β*2-glycoprotein I (*β*2GPI), which is also known as apolipoprotein H, is a phospholipid-binding plasma protein that circulates at a concentration of approximately 4*μ*M. *β*2GPI is composed of five domains and four glycosylation sites connected to the N-terminal of the protein with a “hook-like” crystal structure [6]. Each of domains I to IV has two disulfide bridges, whereas domain V has three disulfide bridges, and the domain V disulfide bond is susceptible to cleavage by oxidoreductases thioredoxin I (TRX-1) and protein disulfide isomerase (PDI) leading to the generation of free thiols at Cys288 and Cys326 [7–9]. This special form of *β*2GPI is called the reduced *β*2GPI which is also the main form of *β*2GPI in plasm [7]. In recent years, this special form of *β*2GPI was reported to play a protective part in diabetic complications. It has been identified that reduced *β*2GPI could inhibit human umbilical endothelial vein cell death induced by H_2_O_2_ [9], indicating it has antioxidative stress effect. This special protein was also found to inhibit the macrophage form foam cell induced by ox-LDL [10] in vitro and inhibit vascular lipid deposition and plaque formation by reducing MMPs/TIMPs expression via the downregulation of the p38MAPK signaling pathway in vivo [11] suggesting that reduced *β*2GPI may alleviate vascular lipid toxicity through anti-inflammation mechanism. Reduced beta2-GPI's anti-inflammatory activity was recently confirmed by our previous study in LPS mediated system inflammation in mice [12]. Our pervious work also made verification that *β*2GPI and reduced *β*2GPI improved renal dysfunction and kidney fibrosis and decreased collagen IV and TGF-*β*1 mRNA and protein expression in STZ-induced diabetic mice and high glucose-induced rat mesangial cells. Moreover, the present studies demonstrated that the renoprotective and antifibrosis effects of *β*2GPI and reduced *β*2GPI in DN were closely associated with suppressing the activation of the TGF-*β*1-p38 MAPK pathway [13].

These pieces of evidence suggest reduced *β*2GPI could play a protective role on diabetic complications through its antioxidative stress activity and anti-inflammation effect, while VEGF-NO axis uncoupling could cause oxidative stress and inflammation. In this present study, we identified whether R-*β*2GPI could play a protective part in the injury of glomerular mesangial cells mediated by high glucose through being involved in the mechanisms of VEGF-NO axis uncoupling.

## 2. Materials and Methods

### 2.1. Cells and Reagents

Rat glomerular mesangial cell line (HBZY-1) and BCA Protein Quantitation kit were purchased from Wuhan Boster Bioengineering company. *β*2GPI was extracted from human blood plasma as previously reported [14] and reduced *β*2GPI was made as in a previous study [14]. The reduced *β*2GPI shows stability and immunoreactivity for at least 24h [14]. Human plasma was purchased from Tianjin municipal blood center (Tianjin, China). Nitric Oxide Assay Kit and Reactive Oxygen Species Assay Kit were purchased from Nanjing Jiancheng Bioengineering Institute (Nanjing Jiancheng Bioengineering Institute, China). First-Strand cDNA Synthesis Kit was purchased from Thermo Scientific (Waltham, MA, USA). RT-PCR kit was purchased from Takara (Japan). The primers were synthetized by Beijing Aoke Bioengineering Institute (Beijing, China.). Rabbit anti-rat eNOS polyclonal antibody and Rabbit anti-rat eNOS Ser1177 polyclonal antibody were purchased from Proteintech Group (Chicago, IL, USA). Rabbit anti-rat Akt polyclonal antibody and Rabbit anti-rat p-Akt polyclonal antibody were purchased from Cell Signaling Technology (Danvers, MA, USA). TRIzol Reagent was purchased from Invitrogen (Carlsbad, CA, US). Tetrahydrobiopterin (BH4) was purchased from Sigma (Sigma-Aldrich Inc., St Louis, MO). This study was approved by the Animal Ethics Committee of the Metabolism Disease Hospital of Tianjin Medical University.

### 2.2. Cell Culture and Treatment

Rat glomerular mesangial cell line was rapidly revived in the constant water bath at 37°C and was cultured in a humidified atmosphere containing 5% CO_2_ in a complete medium composed of DMEM supplemented with 10% fetal bovine serum, with low concentration of glucose. When cells were in the logarithmic phase, they were seeded in sex-well, twelve-well, and ninety-six-well plates. After 24 hours of seeding, rat glomerular mesangial cells were divided into six groups as follows: (a) normal control group with RMCs cultured in 5.5Mm glucose DMEM; (b) osmotic control group with RMCs cultured in DMEM composed of 19.5 mM mannitol and 5.5 mM glucose; (c) high glucose group with RMCs cultured in 25mM glucose DMEM; (d) HSA control group with RMCs cultured in 25mM glucose DMEM supplement with 100*μ*g/ml HSA; (e) RMCs were cultured in 25mM DMEM supplemented with 100*μ*g/ml *β*2GPI; (f) RMCs were cultured in 25mM DMEM supplemented with 100*μ*g/ml r-*β*2GPI or 10 *μ*g/ml BH4. All six groups were incubated for 24 hours in a humidified atmosphere (5% CO_2_ and 37°C).

### 2.3. Measurement of Nitric Oxide and Reactive Oxygen Species

RMCs were plated in six-well plates (2ml/each well). After being incubated for 24 hours, cells were treated with serum-free DMEM for 12 hours. When treated with different intervention, the level of NO was measured as per manufacture's instruction. The microplate reader was used to test the value of OD in the 550nm.

RMCs were cultured as above. After incubation, DCFH-DA, diluted in PBS, was added to each well. According to the wavelength of FITC, the cells were detected and analyzed by flow cytometry to measure intracellular ROS level.

### 2.4. Real-Time Quantitative PCR (RT-qPCR) Analysis

Total RNA was extracted from RMCs treated by interference using TRIzol. The purity and concentration of diluted RNA were measured by spectrophotometry. Gel Imaging System was used to testify the results of electrophoresis to identify the integrity of RNA. First-Strand cDNA was synthesized using 1^st^-Strand cDNA Synthesis Kit. Quantitative real-time PCR assay was prepared by using SYBR Green Mix with the following primers: VEGF (F), 5′GGA GTA CCC CGA TGA GAT AGA 3′; VEGF (R), 5′ GCT GGC TTT GGT GAG GTT TGA3′; VEGFR2 (F), 5′ GAC CGG CTG AAA CTA GGA AAA3′; VEGFR2 (R), 5′ GGA TCT TGA GTT CGG ACA TGA3′; eNOS (F), 5′ CGA CAT TGA GAT CAA AGG ACT G3′; eNOS (R), 5′ ACT TGT CCA AAC ACT CCA CGC3′; GCH-1 (F), 5′ GGC CGC TTA CTC GTC CAT CCT3′; GCH-1 (R), 5′ GGT CTC CTG GTA TCC CTT GGT GAA3′; *β*-actin (F), 5′CCG CAT CCT CTT CCT CCC T3′; *β*-action (R), 5′GCC ACA GGA TTC CAT ACC CAG3′. Expression levels of genes were calculated with 2 ^–ΔΔCt^ method using *β*-actin as internal control genes.

### 2.5. Western Blot Assays

Western blotting analysis was performed to evaluate the activity of eNOS and signaling pathways downstream of VEGF receptors. After 24 hours for intervention, cells were washed twice with ice-cold PBS and were lysed in RIPA buffer containing protease inhibitors for thirty minutes to obtain total proteins' extraction. Samples were added to the automatic microplate reader to test the absorption in A562, and then the concentration in each sample was measured according to standard curve by bicinchoninic acid (BCA) assay. Twenty micrograms of protein extracts was subjected to SDS-PAGE, transferred to nitrocellulose membranes. After being blocked by 5% skimmed milk, the membranes were incubated overnight at 4°C with the primary antibody. After being washed by 1X TBST, the membranes were incubated with corresponding second antibodies at room temperature for one hour. Immunoblots were performed using ECL detection kit and the grey values of bands were measured. To assess eNOS monomer and dimer forms, a loading dye containing *β*-mercaptoethanol was added to unboiled cell lysate and 7.5%native PAGE, and subsequent procedures were performed as previously described [15].

### 2.6. Statistical Analysis

All statistical analyses were performed by using SPSS version 20. The values were presented as mean±SD. For two-group comparison, Independent-Samples T-Test was used. And Least-Significant Difference was performed to compare multiple groups. P value <0.05 was considered statistically significant.

## 3. Results

### 3.1. Reduced *β*2GPI Could Improve the Function of VEGF-NO Axis of HBZY-1 Cells upon High Glucose Treatment

**Figure 1:**
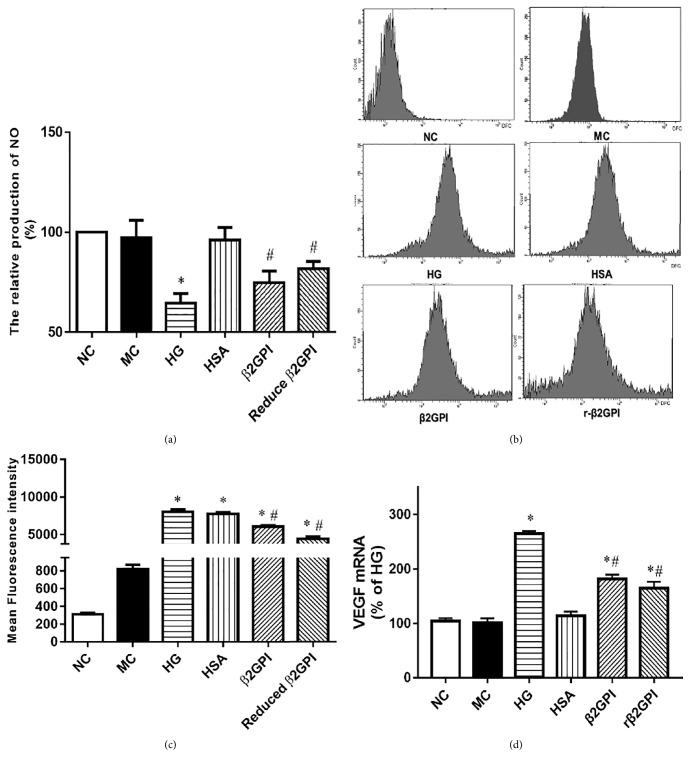
*The effect of reduced β2GPI on the VEGF-NO axis of HBZY-1 cells treated with high glucose.* HBZY-1 cells were exposed to normal glucose, high glucose, or *β*2GPI. (a) The relative production of NO was qualified by using Nitric Oxide Assay Kit. (b-c) The flow cytometry analysis and mean fluorescence intensity of ROS in HBZY-1 cells exposed to different conditions for the indicated time. (d) VEGF mRNA levels were determined by real-time PCR in six disparate experiments. Data are represented as mean ± SD (n=6). ^*∗*^P<0.05 versus NC. ^#^P<0.05 versus HG.

In order to investigate the effect of reduced *β*2GPI on VEGF-NO axis, the production of NO and ROS and the express of VEGF mRNA levels by HBZY-1 cells upon high glucose stimulation were quantified, respectively. As expected, the results in this present study show that high glucose could significantly reduce the production of NO and the VEGF mRNA level and both *β*2GPI and reduced *β*2GPI could partly restore the production of NO and the expression of VEGF mRNA level by HBZY-1 cells treated by high glucose, as shown in Figures 1(a) and 1(d), while the ROS production was assessed by fluorescence probe of DCFH-DA and flow cytometer, respectively. The fluorescence intensity was increased in high glucose group than in control group suggesting high glucose could increase the ROS production by HBZY-1 cells, and both *β*2GPI and reduced *β*2GPI could decrease this effect by high glucose, as shown in Figures 1(b) and 1(c). The quantitative mean fluorescence intensity of Figure 1(c) showed that there was no significant difference between high glucose group, high isotonic group, and HSA group (all P<0.05). High glucose could significantly promote ROS production. And *β*2GPI and r-*β*2GPI could significantly alleviate this effect of high glucose. And this effect was even more obvious in reduced *β*2GPI group, as shown in Figure 1(c).

### 3.2. Reduced *β*2GPI Could Regulate the mRNA Expression Levels of Key Molecules Involved in VEGF-NO Axis Uncoupling in HBZY-1 Cells Induced by High Glucose

VEGFR-2, GCH-1, and eNOS were the key molecules involved in VEGF-NO axis [16, 17]. To further explore the potential mechanism of reduced *β*2GPI participating in VEGF-NO axis uncoupling, the quantitative real-time PCR was performed to measure the mRNA levels of the key members mentioned above expressed by HBZY-1 cells upon high glucose stimulation.

**Figure 2:**
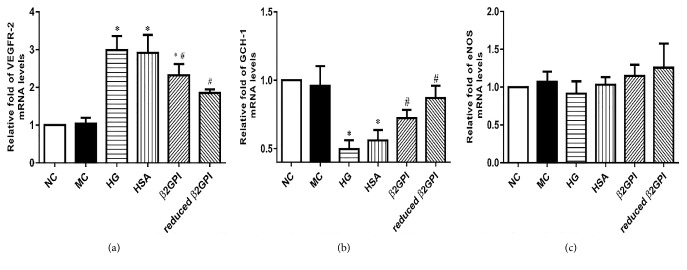
*The effect of reduced β2GPI on the mRNA expression of VEGFR-2, GCH-1 and eNOS of HBZY-1 cells treated with high glucose.* HBZY-1 cells were incubated with normal glucose, high glucose, or *β*2GPI. (a–c) The relative fold of VEGFR-2, GCH-1 and eNOS, and mRNA levels. Data are represented as mean ± SD (n=6). ^*∗*^P<0.05 versus NC. ^#^P<0.05 versus HG.

High glucose could significantly increase the mRNA expression levels of VEGFR-2 and significantly inhibit the expression of GCH-1 mRNA levels, while high glucose had no significant effect on the expression of eNOS expression. Both *β*2GPI and reduced *β*2GPI could significantly reverse these effects of high glucose on HBZY-1 cells. And reduced *β*2GPI had even stronger effect, as shown in Figure 2.

### 3.3. Reduced *β*2GPI Alleviated the Aberrant Phosphorylation of eNOS in HBZY-1 Cells Caused by High Glucose

**Figure 3:**
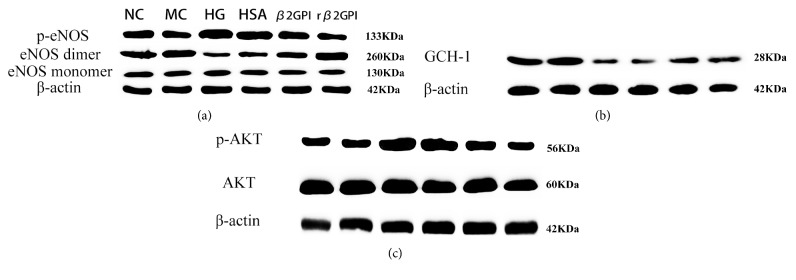
*The effect of reduced β2GPI on key protein in VEGF-NO axis signaling pathway of HBZY-1 cells treated with high glucose.* Protein relative expression of p-AKT, AKT, eNOS dimer, eNOS monomer, p-eNOS, GCH-1, and *β*-actin by western blot after being incubated with different conditions.

The endothelial nitric oxide synthase (eNOS) is a key enzyme and the phosphorylated eNOS could form the dimer which is the active form of eNOS catalyzing the production of NO [15], while high glucose had no significant effect on its mRNA expression as shown above. To further explore the potential mechanism, Western Blot was performed to measure the ration of eNOS dimer-to monomer. The results in this present study showed that the phosphorylation of eNOS (Ser1177) was significantly increased and eNOS dimer is significantly decreased in high glucose group than those in normal group and high isotonic group (P<0.05). And, as expected, both *β*2GPI and reduced *β*2GPI could significantly restore the eNOS dimer-to-monomer ratio. And reduced *β*2GPI had even stronger effect than *β*2GPI, as shown in Figure 3(a). Tetrahydrobiopterin (BH4) is a cofactor that regulates eNOS activity in part by stabilizing the eNOS dimer and promoting eNOS coupling [18].

GCH-1 is one of the key syntheses catalyzing the synthesis of BH4, so this study further assessed the GCH-1 protein levels. The results showed that high glucose could significantly inhibit the expression of GCH-1 levels, both *β*2GPI and reduced *β*2GPI could significantly restore GCH-1 levels, and this effect was even more obvious in reduced*β*2GPI, as shown in Figure 3(b).

Akt signaling pathway, which is the downstream of VEGF receptor, as consistent with previous study [18], high glucose could significantly increase the phosphorylation of Akt, and both *β*2GPI and reduced *β*2GPI could decrease this effect, which was consistent with our previous study [19], as shown in Figure 3(c).

### 3.4. BH4 Partly Reverses the Dysfunction of eNOS Uncoupling Induced by High Glucose

**Figure 4:**
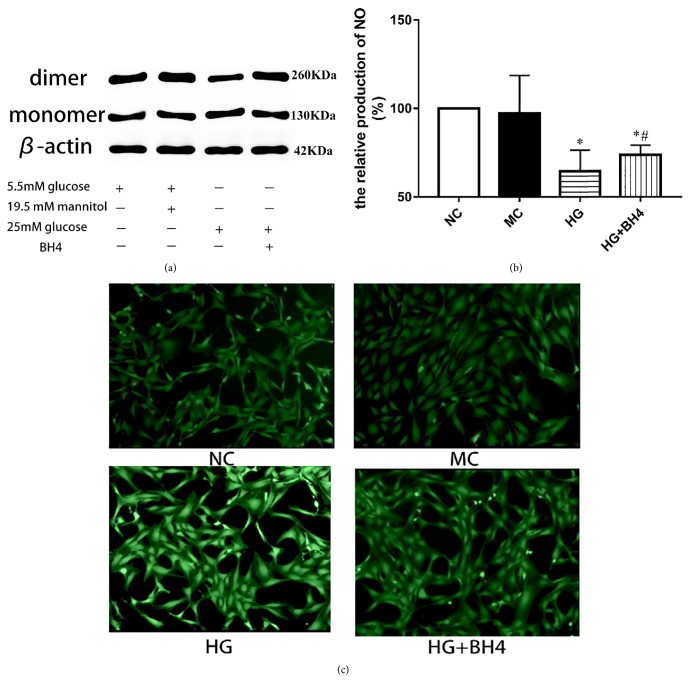
*The Effect of BH4 on eNOS uncoupling.* (a) Protein relative expression of eNOS dimer and eNOS monomer by western blot in four different experiments. (b) The relative production of NO was qualified by using Nitric Oxide Assay Kit. (c) The ROS intensity of immunofluorescence incubated in different experiments. Data are represented as mean ± SD (n=6). ^*∗*^P<0.05 versus NC. ^#^P<0.05 versus HG.

BH4 is the key part of eNOS cofactors, participating in the dimer formation. High glucose could significantly inhibit this procession. In this present study BH4 was added to further reveal this mechanism of eNOS uncoupling. The results showed that BH4 could significantly restore the eNOS dimer levels (Figure 4(a)) and decrease the ROS expression (Figure 4(c)). And the relative expression of NO may also be affected by BH4 (Figure 4(b)).

## 4. Discussion

Diabetic nephropathy, the one of the main causes of the death of diabetic patients, has a specific pathologic characteristic with higher expression of VEGF and lower production of NO in kidney, which is called VEGF-NO axis uncoupling. Both anti-VEGF-NO axis uncoupling or anti-VEGF treatment showed a benefit effect on diabetic nephropathy in mice [4]. A mounting piece of evidence revealed that *β*2GPI played important part in angiogenesis through downregulating VEGF signaling pathway [20] indicating *β*2GPI has the potential effect on diabetic nephropathy. Hyperglycemia is one of the pathogeneses of the high levels of VEGF in diabetic kidney. In this present study, high glucose could significantly increase the expressions of VEGF and VEGFR-2 and upregulated its downstream Akt phosphorylation in glomerular mesangial cells, which is consistent with previous study. The phosphorylated Akt could increase the production of NO [21]. However, NO is significantly reduced in high glucose treated glomerular mesangial cells, indicating the fact that high-glucose treatment caused glomerular mesangial cells VEGF-NO axis uncoupled, which coincides with previous research [20, 22].

As expected, *β*2GPI significantly reduced the expressions of VEGF and VEGFR-2 and downregulated their downstream Akt phosphorylation and showed the anti-VEGF activity as previously reported. However, in this present study, we found that *β*2GPI could partly restore the production of NO by glomerular mesangial cells upon high glucose stimulation. So, we could conclude that high glucose could cause VEGF-NO axis uncoupling in glomerular mesangial cells, and *β*2GPI had the effect of anti-VEGF-NO uncoupling induced by high glucose. And, in this study, we also identified that reduced *β*2GPI had even stronger effect on VEGF-NO axis uncoupling in glomerular mesangial cells induced by high glucose. So, reduced *β*2GPI may be the more active form of *β*2GPI.

As one of critical signal molecules to regulate the activity of eNOS, VEGF achieved this function by mainly recognizing and being combined to VEGFR-2 via downstream signaling, such as PI3K/Akt [23]. Phillip J. White et al. have reported that the Akt could promote the phosphorylation of eNOS to further improve the production of eNOS. In this present study we had found that, under the condition of high concentration glucose, the expressions of VEGF and its receptor VEGFR-2 were higher than normal control, and the phosphorylation level of Akt was also significantly higher than control which were consistent with previous study [20]. The activation and inactivation of eNOS were mainly regulated by the phosphorylation and dephosphorylation. The phosphorylated eNOS could form eNOS dimer which catalyzes NO production [15], while the level of eNOS monomer increase could lead to ROS production called eNOS alternative pathway [24].

Although high glucose could promote VEGF production and its downstream signaling protein Akt phosphorylation, the activity of eNOS seemed decreased, indicating that eNOS dimer formation dysfunction could partly explain the VEGF-NO axis uncoupling in diabetic nephropathy. In this study, we found that high glucose could increase ROS production also indicating eNOS monomer production increased and eNOS alternative pathway increased by high glucose.

Tetrahydrobiopterin (BH4) is the key part of eNOS cofactors, participating in eNOS dimers formation, the eNOS activity form. GTP cyclohydrolase 1 (GCH-1) is the speed limit of BH4 synthesis enzyme [25]. In this present study, we assess the levels of GCH-1 mRNA and protein levels, and the result showed that high glucose could significantly inhibit the expression of GCH-1 indicating that the decrease of GCH-1 may be one of the mechanisms of eNOS dimers formation dysfunction caused by high glucose. To further identify this mechanism, we performed the BH4 experiment. The result in Figure 4 showed that BH4 could increase the eNOS dimers formation and decrease ROS production. Based on these results we concluded that *β*2GPI and reduced *β*2GPI could significantly increase GCH-1 expression then further to promote the eNOS dimer formation and the production of NO. On the other hand, eNOS monomer and ROS production decreasing could alleviate the oxidative stress, which at lease could partly explain reduced *β*2GPI has the antioxidative stress activity.

In conclusion, high glucose could cause the dysfunction of eNOS dimer formation in HBZY-1 leading to VEGF-axis uncoupling. *β*2GPI and r-*β*2GPI can partly reverse the VEGF-NO axis uncoupling induced by high concentration glucose in glomerular mesangial cells through promoting eNOS dimer formation. Reduced *β*2GPI is the more active form of *β*2GPI.

## Abbreviations

*β*2GPI: *β*2-glycoprotein I
VEGF: Vascular endothelial growth factor
NO: Nitric oxide
eNOS: Endothelial nitric oxide synthase
BH4: Tetrahydrobiopterin
GCH-1: GTP cyclohydrolase 1.

## References

[1] Packham D. K., Alves T. P., Dwyer J. P. (2012). Relative incidence of ESRD versus cardiovascular mortality in proteinuric type 2 diabetes and nephropathy: results from the DIAMETRIC (Diabetes Mellitus Treatment for Renal Insufficiency Consortium) database. *American Journal of Kidney Diseases*.

[2] Fruchart J.-C., Sacks F. M., Hermans M. P. (2008). The residual risk reduction initiative: a call to action to reduce residual vascular risk in dyslipidaemic patients. *Diabetes & Vascular Disease Research*.

[3] Nakagawa T., Sato W., Sautin Y. Y. (2006). Uncoupling of vascular endothelial growth factor with nitric oxide as a mechanism for diabetic vasculopathy. *Journal of the American Society of Nephrology*.

[4] Nakagawa T., Sato W., Kosugi T., Johnson R. J. (2013). Uncoupling of VEGF with Endothelial NO as a Potential Mechanism for Abnormal Angiogenesis in the Diabetic Nephropathy. *Journal of Diabetes Research*.

[5] Nakagawa T. (2008). Uncoupling of VEGF with NO as a mechanism for diabetic nephropathy. *Diabetes Research and Clinical Practice*.

[6] Miyakis S., Giannakopoulos B., Krilis S. A. (2004). Beta 2 glycoprotein I-function in health and disease. *Thrombosis Research*.

[7] Passam F. H., Rahgozar S., Qi M. (2010). Beta 2 glycoprotein I is a substrate of thiol oxidoreductases. *Blood*.

[8] Passam F. H., Rahgozar S., Qi M. (2010). Redox control of *β*2-glycoprotein I-von Willebrand factor interaction by thioredoxin-1. *Journal of Thrombosis and Haemostasis*.

[9] Ioannou Y., Zhang J.-Y., Passam F. H. (2010). Naturally occurring free thiols within *β*2-glycoprotein I in vivo: nitrosylation, redox modification by endothelial cells, and regulation of oxidative stress-induced cell injury. *Blood*.

[10] Wang W.-L., Meng Z.-X., Zhou S.-J. (2013). Reduced beta2-glycoprotein i protects macrophages from ox-LDL-induced foam cell formation and cell apoptosis. *Lipids in Health and Disease*.

[11] Xu J., Wang P., Wang T. (2014). Effects of reduced *β*2-glycoprotein I on the expression of aortic matrix metalloproteinases and tissue inhibitor matrix metalloproteinases in diabetic mice. *BMC Cardiovascular Disorders*.

[12] Zhou S., Chen G., Qi M. (2016). Gram negative bacterial inflammation ameliorated by the plasma protein beta 2-Glycoprotein I. *Scientific Reports*.

[13] Wang T., Chen S. S., Chen R., Yu D. M., Yu P. (2018). Reduced beta 2 glycoprotein I improves diabetic nephropathy via inhibiting TGF-beta1-p38 MAPK pathway. *International journal of clinical and experimental pathology*.

[14] Zhou S., Lu M., Zhao J. (2017). The purification of reduced beta2-glycoprotein I showed its native activity in vitro. *Lipids in Health and Disease*.

[15] Chang C.-C., Hsu Y.-H., Chou H.-C., Lee Y.-C. G., Juan S.-H. (2017). 3-Methylcholanthrene/Aryl-Hydrocarbon Receptor-Mediated Hypertension Through eNOS Inactivation. *Journal of Cellular Physiology*.

[16] Sugiyama T., Levy B. D., Michel T. (2009). Tetrahydrobiopterin recycling, a key determinant of endothelial nitric-oxide synthase-dependent signaling pathways in cultured vascular endothelial cells. *The Journal of Biological Chemistry*.

[17] Cai J., Jiang W. G., Ahmed A., Boulton M. (2006). Vascular endothelial growth factor-induced endothelial cell proliferation is regulated by interaction between VEGFR-2, SH-PTP1 and eNOS. *Microvascular Research*.

[18] Jin J., Yuan F., Shen M.-Q., Feng Y.-F., He Q.-L. (2013). Vascular endothelial growth factor regulates primate choroid-retinal endothelial cell proliferation and tube formation through PI3K/Akt and MEK/ERK dependent signaling. *Molecular and Cellular Biochemistry*.

[19] Dikalova A., Aschner J. L., Kaplowitz M. R., Summar M., Fike C. D. (2016). Tetrahydrobiopterin oral therapy recouples eNOS and ameliorates chronic hypoxia-induced pulmonary hypertension in newborn pigs. *American Journal of Physiology-Lung Cellular and Molecular Physiology*.

[20] Liu H., Zhou S., Denyer G. (2015). Reduced beta2glycoprotein capital I, Ukrainian inhibits hypoxiainduced retinal angiogenesis in neonatal mice through the vascular endothelial growth factor pathway. *Molecular Medicine Reports*.

[21] Zhai Y.-P., Lu Q., Liu Y.-W. (2013). Over-production of nitric oxide by oxidative stress-induced activation of the TGF-*β*1/PI3K/Akt pathway in mesangial cells cultured in high glucose. *Acta Pharmacologica Sinica*.

[22] Wang Q., Zhou S., Meng Z. (2015). Domain I-IV of *β*2-glycoprotein I inhibits advanced glycation end product-induced angiogenesis by down-regulating vascular endothelial growth factor 2 signaling. *Molecular Medicine Reports*.

[23] Byeon S. H., Lee S. C., Choi S. H. (2010). Vascular endothelial growth factor as an autocrine survival factor for retinal pigment epithelial cells under oxidative stress via the VEGF-R2/PI3K/Akt. *Investigative Ophthalmology & Visual Science*.

[24] Bauersachs J., Schäfer A. (2005). Tetrahydrobiopterin and eNOS dimer/monomer ratio - A clue to eNOS uncoupling in diabetes?. *Cardiovascular Research*.

[25] Alp N. J., Channon K. M. (2004). Regulation of endothelial nitric oxide synthase by tetrahydrobiopterin in vascular disease. *Arteriosclerosis, Thrombosis, and Vascular Biology*.

[26] Wang J. (2015). *The mechanism of reduced β2GPI inhibiting glomerular mesangial cells VEGF-NO axis uncoupling induced by high glucose [D]*.

